# Supplementary material for: Porcine Circovirus Type 2 Vaccines: Commercial Application and Research Advances
Source: Viruses. 2022 Sep 10;14(9):2005. doi: 10.3390/v14092005 (PMC9504358; doi:10.3390/v14092005)
Supplement: Supplementary file 1 [file viruses-14-02005-s001.zip › viruses-1908339-supplementary.pdf]

Table S1. PCV2 vaccines marketed in China

Type of vaccine	PCV2 strain	Product name	Animals	Dosage	Administration	Duration of	Virus content per mL	Production enterprise
PCV2 inactivated vaccine	Strain SH	Yuan jian	Piglet	One (1 mL)	2-3 weeks+2 weeks interval	4	10^6.0 $TCID_{50}$	Pulike Biological Engineering Co., Ltd.
PCV2 inactivated vaccine	Strain SH		Piglet	One (1 mL)	2-3 weeks+2 weeks interval	4	10^6.0 $TCID_{50}$	Jilin Zhengye Biological Products Co., Ltd.
PCV2 inactivated vaccine	Strain SH	Yuan hao jia	Piglet	One (1 mL)	2-3 weeks+2 weeks interval	4	10^6.0 $TCID_{50}$	Qianyuanhao Biological Technology (Nanjing) Co., Ltd.
PCV2 inactivated vaccine	Strain SH	Yuan yi	Piglet	One (1 mL)	2-3 weeks+2 weeks interval	3	10^6.0 $TCID_{50}$	Changzhou Tongtai Biological Pharmaceutical
PCV2 inactivated vaccine	Strain SH	Ao lan yuan qing	Sow/Piglet/Boar	One (sow, boar, 2 mL; piglet, 1 mL)	3-4 weeks (sow, antepartum); 2 weeks (piglet); 3-4 times/year (Boar)	6	10^7.5 $TCID_{50}$	Qingdao Oland Biological Engineering Co., Ltd.
PCV2 inactivated vaccine	Strain SH	Yuan bei an	Piglet	One (1 mL)	2-3 weeks+2 weeks interval	3	10^6.0 $TCID_{50}$	Beijing Huadu Biotechnology Co., Ltd
PCV2 inactivated vaccine	Strain SH	Yuan ke qing	Piglet	One (1 mL)	2-3 weeks+2 weeks interval	3	10^6.0 $TCID_{50}$	Jiangsu Nannong Hi-Tech Co., Ltd.
PCV2 inactivated vaccine	Strain SH, ||	Yuan ke qing jia	Sow/Piglet	One (sow, 4 mL; piglet, 1 mL)	40-45 days (sow, antepartum)+3 weeks interval; 2-4 weeks (piglet)+2 weeks interval	4	10^6.0 $TCID_{50}$	Jiangsu Nannong Hi-Tech Co., Ltd.
PCV2 inactivated vaccine	Strain SH, ||	Yuan meng	Sow/Piglet	One (sow, 4 mL; piglet, 1 mL)	40-45 days (sow, antepartum)+3 weeks interval; 2-4 weeks (piglet)+2 weeks interval	4	10^6.0 $TCID_{50}$	China Animal Husbandry Industry (Chengdu) Co., Ltd.
PCV2 inactivated vaccine	Strain SH, ||	Yuan bei shi	Sow/Piglet	One (sow, 4 mL; piglet, 1 mL)	40-45 days (sow, antepartum)+3 weeks interval; 2-4 weeks (piglet)+2 weeks interval	4	10^6.0 $TCID_{50}$	Luo Yang Huizhong Biotechnology Co., Ltd.
PCV2 inactivated vaccine	ZJ/C	Yuan jing nuo	Piglet	One (2 mL)	2 weeks+	4	10^6.0 $TCID_{50}$	Zhejiang Ebvac Biotechnology Co., Ltd.
PCV2 inactivated vaccine	ZJ/C	Yuan huan kang	Sow/Piglet/Boar	One (sow, boar, piglet, 2 mL)	2 times (sow, before breeding); 2-3 weeks (piglet); 2 times/year (Boar)		10^8.0 $TCID_{50}$-10^8.3 $TCID_{50}$	Huapai Biological Co., Ltd
PCV2 inactivated vaccine	ZJ/C	You yuan bao	Sow/Piglet/Boar	One (sow, boar, 2 mL; piglet, 1 mL)	4 times/year (sow); 3-4 weeks (piglet); 3 times/year (Boar)			Hang Zhou Youben Animal Vaccine Co., Ltd.
PCV2 inactivated vaccine	ZJ/C	Yuan jie	Piglet	One (2 mL)	2 weeks+	4	10^7.3 $TCID_{50}$	Qilu Animal Health Products Co., Ltd.
PCV2 inactivated vaccine	ZJ/C	Zhu yuan tuo	Piglet	One (2 mL)	2 weeks+	4	10^7.3 $TCID_{50}$	Ruipu (Bao ding) Pharmaceutical Co., Ltd.
PCV2 inactivated vaccine	ZJ/C	Zheng yuan jing	Piglet	One (2 mL)	2 weeks+	4	10^7.3 $TCID_{50}$	Guizhou Firstv Biotechnology Co., Ltd.
PCV2 inactivated vaccine	YZ	Yuan li ding	Piglet	One (1 mL)	3 weeks		10^7.0 $TCID_{50}$	Zhejiang Mebolob Biotechnoligy Co., Ltd.
PCV2 inactivated vaccine	YZ	Yuan li ning						Guangxi Liyuan Biological Co., Ltd.
PCV2 inactivated vaccine	YZ	Yuan li jing						Yangzhou Uni-Bio Pharmaceutical Co., Ltd.
PCV2 inactivated vaccine	WH							Wuhan Chopper Biology Co., Ltd.
PCV2 inactivated vaccine	WH	Yuan man	Piglet	One (2 mL)	3-4 weeks	3	10^7.0 $TCID_{50}$	China Animal Husbandry Industry (Chengdu) Co., Ltd.
PCV2 inactivated vaccine	WH	Ke yuan ning	Piglet	One (3 mL)	3-5 weeks	3	10^7.0 $TCID_{50}$	Wuhan Keqian Biological Co., Ltd.
PCV2 inactivated vaccine	WH	Yuan bi ning	Sow/Piglet	One (2 mL)	3 times/year (sow); 2-3 weeks (piglet)		10^7.0 $TCID_{50}$	Zhaofenghua Biotechnology Co., Ltd
PCV2 inactivated vaccine	WH		Piglet	One (2 mL)	3-4 weeks	3	10^7.0 $TCID_{50}$	Guangdong Winsun Pharmaceutical Co., Ltd.
PCV2 inactivated vaccine	DBN-SX07	Hai yuan an	Piglet	One (1 mL)	2-3 weeks+2 weeks interval	4	10^5.5 $TCID_{50}$	Sichuanhailing Biological Pharmaceutical Co., Ltd.
PCV2 inactivated vaccine	DBN-SX07	Dong fang yuan		One (1 mL)	2-3 weeks+2 weeks interval	4	10^5.5 $TCID_{50}$	Anhui Divinity Biological Products Co., Ltd
PCV2 inactivated vaccine	DBN-SX07	Yuan li jia	Piglet	One (1 mL)	2-3 weeks+2 weeks interval	4	10^5.5 $TCID_{50}$	Chengdu Tecbond Biological Products Co., Ltd
PCV2 inactivated vaccine	DBN-SX07	Zhu huan tai	Piglet	One (1 mL)	2-3 weeks+2 weeks interval	4	10^5.5 $TCID_{50}$	Zhaofenghua Biotechnology Co., Ltd
PCV2 inactivated vaccine	DBN-SX07	Xin yuan ning	Sow/Boar	One (1 portion)	3-4 times/year (sow, Boar); 2-3 weeks (piglet)+2 weeks interval	4		Bejing Sinder Biological Technological Co., Ltd
PCV2 inactivated vaccine	DBN-SX07	Yuan kang	Piglet	One (1 mL)	2-3 weeks+2 weeks interval	4	10^5.5 $TCID_{50}$	Liaoning Yikang Biological Co., Ltd
PCV2 inactivated vaccine	DBN-SX07							Shandong Huahong Biological Products Co., Ltd
PCV2 inactivated vaccine	DBN-SX07	lv yuan kang	Piglet	One (1 mL)	2-3 weeks+2 weeks interval		10^5.5 $TCID_{50}$	Shandong Lvdubio-Science& Technology Co., Ltd.
PCV2 inactivated vaccine	DBN-SX07	Wo yuan qing	Sow/Piglet/Boar	One (2 mL)	3 times/year (sow); 2-3 weeks (piglet); 2 times/year (Boar)	4		Shandong Binzhou Wohua Biological Engineering Co.,
PCV2 inactivated vaccine	DBN-SX07	Yuan zhi you	Sow/Piglet/Boar	One (sow, boar, 2 mL; piglet, 1 mL)	3-4 times/year (sow, Boar); 2-3 weeks (piglet)+2 weeks interval	4	10^5.5 $TCID_{50}$	Hunan Sinoland Biological Pharmaceutical Co., Ltd.
PCV2 inactivated vaccine	LG	Yuan bi jing	Sow/Piglet/Boar	One (sow, boar, 2 mL; piglet, 1 mL)	2 times (sow, before breeding)+1 times (sow, antepartum); 3-4 weeks (piglet)+3 weeks interval; 3 times/year (Boar)		10^5.5 $TCID_{50}$	Shanghai Hile Biological Technology Co., Ltd
PCV2 inactivated vaccine	LG	Yuan mei rui	Sow/Piglet/Boar	One (sow, boar, 2 mL; piglet, 1 mL)	2 times (sow, before breeding)+1 times (sow, antepartum); 3-4 weeks (piglet)+3 weeks interval; 3 times/year (Boar)		10^5.5 $TCID_{50}$	Harbin Pharmaceutical Group Bio-Vaccine Co., Ltd
PCV2 inactivated vaccine	LG	Zhu yuan kang	Sow/Piglet	One (sow, 2 mL; piglet, 1 mL)	4-5 weeks (sow, antepartum); 2 weeks (piglet)		10^6.5 $TCID_{50}$	Qingdao Vland Biological Technology Co., Ltd.
PCV2 inactivated vaccine	LG							Harbin Weike Bintechnology Co., Ltd.
Subunit vaccine		Yi yuan jing	Sow/Piglet/Boar	One (2 mL)	1 times (sow, before breeding); 2-4 weeks (piglet); 3 times/year (Boar)	4	Cap protein content exceeds 100 μg/mL	Qingdao Ye Biological Enginering Co., Ltd.
Subunit vaccine		Yuan ke xin	Sow/Piglet	One (sow, 2 mL; piglet, 1 mL)	4-5 weeks (sow, antepartum); 2-4 weeks (piglet)	4	Cap protein content exceeds 40 μg/mL	Pulike Biological Engineering Co., Ltd.
Vectored vaccine	CP08	Yuan huan li kang	Sow/Piglet/Boar	One (1 mL)	3 times/year (sow, Boar); 2-3 weeks (piglet)	4		Wuhan Chopper Biology Co., Ltd.
Vectored vaccine	CP08	Yuan li you	Sow/Piglet	One (1 mL)	3 weeks (sow, before breeding) +5-6 weeks (sow, antepartum); 2-3 weeks (piglet)	4		Yangzhou Uni-Bio Pharmaceutical Co., Ltd.
